# Coupling ultrasound and membrane filtration for the fractionation of *Spirulina platensis sp.* and the recovery of phycocyanin and pigment-free proteins

**DOI:** 10.1007/s10529-024-03541-9

**Published:** 2024-11-29

**Authors:** Sara Obeid, Hussein Rida, Jérôme Peydecastaing, Hosni Takache, Ali Ismail, Pierre-Yves Pontalier

**Affiliations:** 1 Laboratoire de Chimie Agro-Industrielle (LCA), Université de Toulouse, Toulouse INP, INRAE France; 2 Département des Sciences et Technologies Alimentaires, Faculté d’Agronomie, Université Libanaise, Dekwaneh, Lebanon; 3 Plateforme de Recherche et d’Analyses en Sciences de L’Environnement (PRASE), Ecole Doctorale des Sciences et Technologies, Université Libanaise, Hadath, Lebanon

**Keywords:** Membrane process, Microalgae, Phycocyanin, Protein purification, Sonoextraction

## Abstract

The cyanobacterium *Spirulina platensis* was subjected to a fractionation process involving ultrasound-assisted extraction and membrane filtration to obtain a pure phycocyanin fraction and a clarified colorless protein fraction free of chlorophyll and carotenoids. The effects of pressure and power on total protein release were assessed. The retention of the extracted proteins was then assessed by ultrafiltration, with and without ammonium sulfate precipitation. Total protein recovery yields reached 97% in aqueous solution, at a low frequency (12 kHz), atmospheric pressure, and with an ultrasonic power of 200 Watts (W). Ammonium sulfate (25% w/v) precipitation was used to remove pigments and impurities from the crude protein extract. Finally, semi-frontal ultrafiltration resulted in high levels of C-phycocyanin recovery in the retentate: 95% and 91% with 10 and 100 kDa-cutoff membranes, respectively. However, the levels of total non-pigmented proteins in the permeate compartment did not exceed 67% with a 100 kDa-cutoff membrane. A fractionation process is proposed here for the valorization of two different protein fractions from *Spirulina platensis*.

## Introduction

*Spirulina platensis* is a cyanobacterium widely used as a source of proteins for human consumption due to its high abundance of proteins and essential amino acids (AlFadhly et al. 2022). The protein content of these edible blue-green algae may reach 70% of total dry biomass, exceeding that of common plant or animal protein sources, such as soybean (35%), groundnut (25%), meat and fish (15–25%), eggs (12%), and whole milk (3%) (Fernandes et al. 2023).

Phycobiliproteins are water-soluble proteins that play an important role in harvesting light energy from sunlight and transferring it to the photosynthetic reaction center, which contains a special pair of chlorophyll molecules. These colored phycobiliproteins are found in covalently bound form to thylakoid system and its photosynthetic lamellae. *Spirulina* produces several different phycobiliproteins, including allophycocyanin (APC), C-phycocyanin (C–PC), and phycoerythrin (PE). The principal phycobiliprotein fraction consists of C–PC, which may account for approximately 20% of the total dry biomass (Pez Jaeschke et al. 2021; Dagnino-Leone et al. 2022)*.*

C–PC is used commercially as a natural pigment in the food and cosmetic industries but it also has various uses in the field of nanotechnology (Ashaolu et al. 2021). Non-pigmented protein fractions are increasingly being explored for pharmaceutical and cosmetic uses, but their potential is often limited by the presence of colored compounds, which affect color and flavor (Grossmann et al. 2020). The potential uses of *Spirulina* proteins could, therefore, be increased by removing C–PC, APC, and other undesirable colored components.

In addition, effective protein recovery from *Spirulina* requires cell disruption to release intracellular proteins (Vernès et al. 2019). Ultrasound-assisted extraction (UAE) has been used in fractionation procedures for microalgae and cyanobacteria, facilitating cell disruption and protein release (Deng et al. 2023). Two technologies were compared here: traditional sonication and mano-thermo-sonication (MTS), which combines pressure and ultrasound to improve extraction yields for intracellular molecules, including phycocyanin and other related biomolecules (Meullemiestre et al. 2017). Low-frequency ultrasound has been shown to be more suitable and efficient for cell destruction (Delran et al. 2023), but the use of ultrasound at audible frequencies (20 Hz–20 kHz) for the extraction of proteins from microalgae has never been reported. This study investigated the use of gentle ultrasound-assisted extraction at 12 kHz, a frequency previously unexplored for *Spirulina.*

*Spirulina* has a particularly fragile cellular wall and UAE can release a complex mixture of components, necessitating challenging and costly downstream purification processes (Martínez-Sanz et al. 2020; Fabre et al. 2024). Low-purity C–PC can be used as a biocolorant in the food and cosmetics industries, but a higher purity is required for analytical-grade C–PC for therapeutic and biomedicine applications (Figueira et al. 2018). Ultrafiltration (UF) is a separation method for the effective purification and concentration of proteins without the need for thermal denaturation or chemical solvents. UF has been studied for the purification of polysaccharides and the concentration of proteins from microalgae (Zhao et al. 2020; Liu et al. 2021; Costa et al. 2021; Ribeiro et al. 2022). Filtration processes have also been used to recover C–PC from *Spirulina sp.* (Nisticò et al. 2022; Melanie et al. 2023)*.* Phosphate buffer extraction was found to yield C–PC with purity of 0.53, but UF followed by a single diafiltration step with a 50 kDa-cutoff membrane increased purity to 0.76 (Brião et al. 2020).

Most studies to date have focused on the extraction of phycobiliproteins (C–PC), neglecting other colorless proteins. The use of sonication (35 kHz, 20% power, 50% duty cycle, and 7 min of irradiation time) followed by a liquid biphasic system has been reported to extract 95% C–PC from *Spirulina sp.* (Chia et al. 2019). A process for extracting C–PC from *Spirulina* by bead milling has been developed, with purification based on diafiltration with a ceramic membrane with a transmembrane pressure (TMP) of 4 bar and a 300 kDa molecular weight cutoff (MWCO) (Balti et al. 2021). A C–PC extraction yield of 2.5 mg C–PC g^−1^ DW was reported following sonication at a frequency of 35 kHz and a power of 300 W for 3 h (Minchev et al. 2020).

The purification of polysaccharides extracted by sonication at 24 kHz has been explored (Zhou et al. 2023), but very few studies have considered the use of ultrasound and ultrafiltration in *Spirulina*. Proteins have been extracted with ultrasound at a frequency of 40 kHz and an amplitude of 90% for 35 min, and then purified with a membrane-based process (Menegotto et al. 2020). Despite the studies on C–PC valorization performed, a critical gap in the literature persists regarding the clarification and valorization of both pigmented and non-pigmented proteins in a single process. This gap is particularly crucial at the industrial scale, as each fraction has different applications. This study therefore explored the use of low-frequency ultrasound (12 kHz) followed by UF for the extraction and purification of non-pigmented proteins and the production of C–PC from fresh *Spirulina sp.* This is the first time that such a low frequency has been used for the rupture of *Spirulina* cells, which adds the novelty to this work.

## Materials and methods

### Biomass and chemicals

*Spirulina platensis* was purchased from Alg&You (Toulouse, France) as fresh biomass. It was cultured in a photobioreactor under normal growth conditions, as recommended by the supplier. Fresh biomass was supplied in a liquid culture medium (concentrated at 5% dry weight). All experiments were conducted within two days of the arrival of the algal suspension, to minimize the potential alterations to the structure of the algae.

Lowry protein assay kits were purchased from Thermo Fisher Scientific (France). All chemicals and standards were of analytical grade and purchased from Sigma-Aldrich (France).

## Cell disruption

**Fig. 1:**
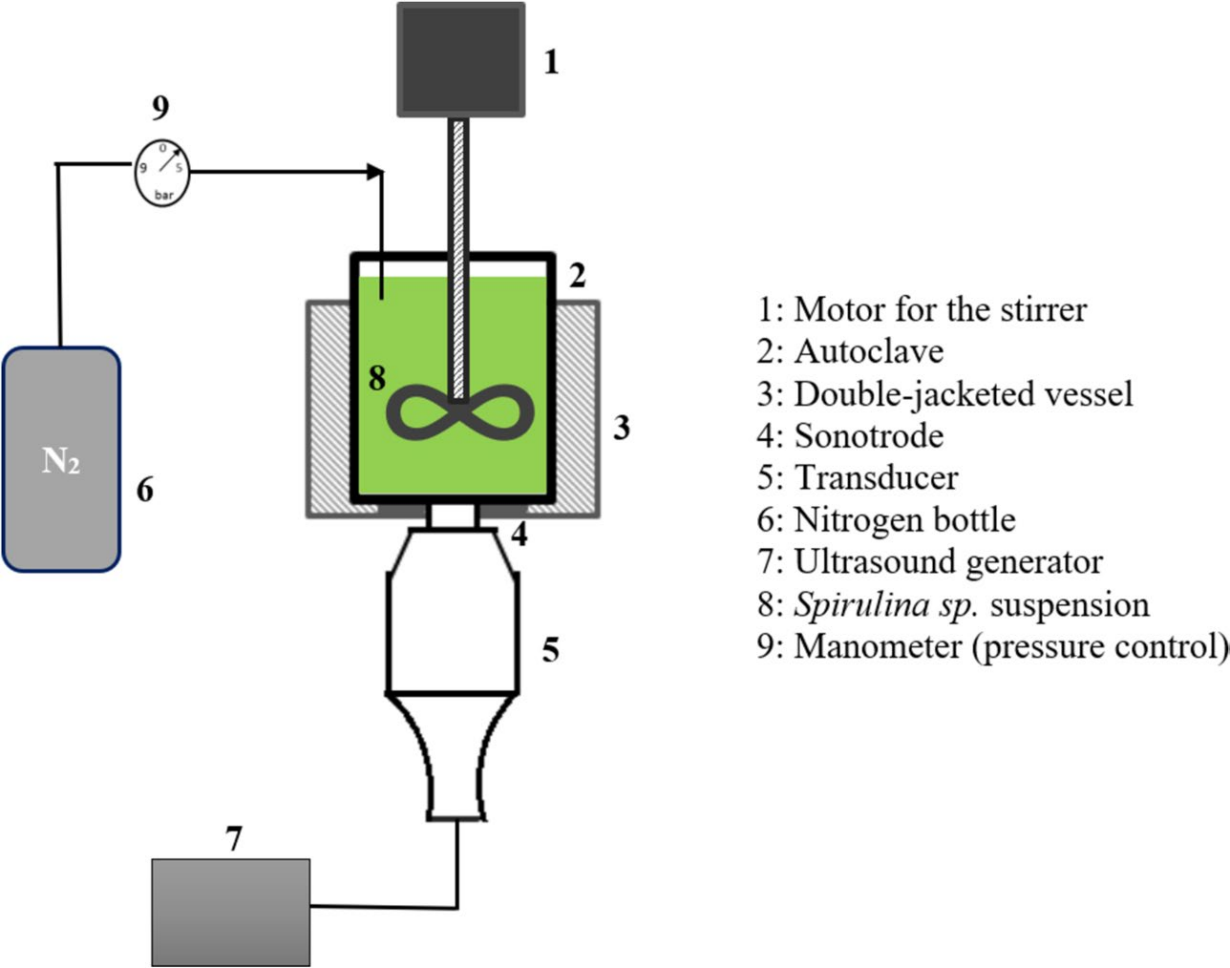
Schematic diagram of the pilot ultrasound apparatus used for cell disruption. 1: Motor for the stirrer, 2: autoclave, 3: double-jacketed vessel, 4: sonotrode, 5: transducer, 6: nitrogen bottle, 7: ultrasound generator, 8: *Spirulina sp.* suspension, 9: Manometer (pressure control)

The algal suspension (600 ml) in aqueous medium was treated by UAE, with water as the solvent. The suspension was placed in a stainless-steel cylinder with a capacity of 1 L and pressurized with nitrogen gas (Fig. 1). The temperature was maintained at 30 ± 2 °C by circulating cooling water through an internal coil to prevent excessive heating. The solution was homogenized by constant stirring with a magnetic bar at 500 rotations per min (rpm) and sonicated at a low frequency (12 kHz). The effects of ultrasound on protein recovery were studied (Table 1), taking the following parameters into account: acoustic power (100 W, 200 W, and 300 W), relative hydrostatic pressure (0 bar, 1.5 bar, and 3 bar), and stirring time (10 min and 60 min). The cells were disrupted and the mixture was centrifuged at 15,000 × *g* for 15 min at 5 °C, and the resulting supernatant was collected for further analyses of protein content.

**Table 1:** Conditions for the disruption of fresh *Spirulina sp.* cells

Experiment code	Time (min)	Relative pressure (bar)	US power (W)	Frequency (kHz)
P0W0	60	0	0	0
P0W100-10	10	0	100	12
P0W100-60	60	0	100	12
P0W200	60	0	200	12
P0W300	60	0	300	12
P1.5W100	60	1.5	100	12
P3W100	60	3	100	12
P3W300	60	3	300	12

*P0* atmospheric pressure, *P1.5* pressure at 1.5 bar, *P3* pressure at 3 bar, *W* power of 0, 100, 200 and 300 W

## Precipitation of the non-protein fraction

The UAE extracts were centrifuged to remove residues. The resulting greenish supernatants were rich in undesirable pigments and lipids and were subjected to precipitation in 25% (w/v) ammonium sulfate. The sample/ammonium sulfate mixture was stored overnight in the dark at 5 °C and was then centrifuged at 15,000 × *g* for 15 min at 5 °C. The supernatant was recovered and subjected to UF to recover non-pigmented proteins free of C–PC and other pigments.

## Ultrafiltration

Semi-frontal ultrafiltration was performed with a laboratory-scale Amicon® stirred-cells UF unit (Thermo Fisher Scientific), with a maximum volume of 200 ml and a filtration area of 30.17 cm^2^. This unit was fitted with a polyethersulfone (PES) disc membrane with a molecular weight cutoff (MWCO) of 10 kDa or 100 kDa. During UF, 100 ml of feeding solution was constantly stirred (200 rpm) in the feeding chamber, minimizing the concentration polarization effect at the membrane surface (Liang et al. 2023). The TMP was kept constant at 2 bar for all experiments. The pigment-free protein fraction was recovered in the permeate compartment. Samples of the permeate and retentate were taken for the analysis of total chlorophylls, total carotenoids, proteins, and C–PC.

**Fig. 2:**
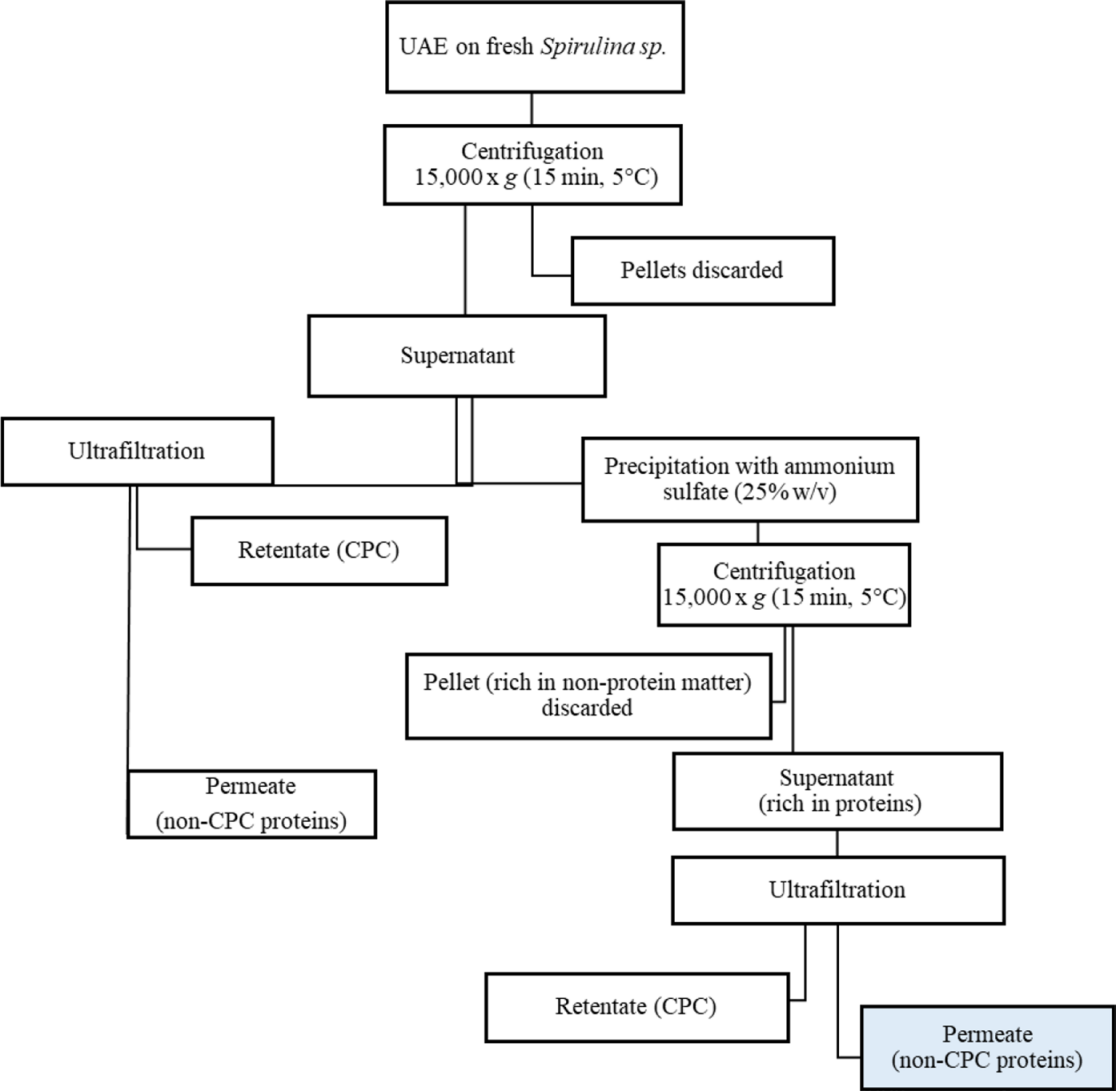
Schematic representation of the process for protein recovery from *Spirulina sp.* studied

Two series of UF procedures were performed:

i) A sample of the supernatant from the experiment with the highest protein extraction yield was subjected to UF to obtain a pure fraction of non-pigmented proteins.
ii) Another sample from the same experiment was subjected to precipitation with 25% (w/v) ammonium sulfate followed by UF (Fig. 2).

## Analysis

### Total proteins

Total protein content was determined by the Kjeldahl method, which uses a coefficient of 6.25 to account for the total nitrogen present in the biomass (Ben Mya et al. 2024). Biomass analysis was performed in triplicate, on 50 mg samples.

## Soluble proteins

The soluble protein content of the UAE supernatants was determined in Lowry assays (Lowry et al. 1951) performed with a Lowry protein assay kit (Lowry reagent plus bovine serum albumin (BSA) standards, and 2 N Folin–Ciocalteu reagent) purchased from Thermo Fisher Scientific (Illkirch, France). Before analysis, the residues were removed from the supernatants by centrifugation at 15,000 × *g* for 15 min. Absorbance was measured at 750 nm with a Shimadzu UV-1800 UV–Vis spectrophotometer.

## APC and C–PC quantification

The C–PC content of the strain used was analyzed after extraction in phosphate buffer (Boussiba and Richmond 1979). C–PC content was determined as the total amount of C–PC and APC. C–PC recovery in UAE supernatant, permeate and retentate was assessed with a Shimadzu UV-1800 UV–Vis spectrophotometer and the following equations (Bennett and Bogorad 1973; Patel et al. 2005):

1 $$APC = \frac{{(OD_{652} - 0.208OD_{620} )}}{5.09}$$

2 $$C - PC = \frac{{(OD_{620} - 0.474OD_{652} )}}{5.34}$$

The retention rate for C–PC was determined as follows (Jaouen et al. 1999):

3 $${\text{Re}} tention\,rate\,(\% ) = 100 - \left[ {1 - \frac{{OD_{620,Permeate} }}{{OD_{{620,{\text{Re}} \tan tate}} }}} \right]$$

## Pigment analysis

Pigment analysis was performed on the supernatant as previously described (Safi et al. 2017). Pigment concentrations were determined with the following equations (Ritchie 2006):

4 $$Total\,chlorophyll\,(\mu gl^{ - 1} ) = (9.3443 \times OD_{652} ) + (4.3481 \times OD_{665} )$$

5 $$Total\,carotenoids\,(\mu gl^{ - 1} ) = 4 \times OD_{480}$$

## Microscopy

The efficiency of cell disruption was assessed by examining a sample of the cell suspension placed on a specific plate under a stereo microscope (Nikon SMZ 1500) before and after disruption. The images were captured at a magnification of × 1000 under constant illumination and exposure, with a Nikon Eclipse E600 camera.

## Statistical analysis

Each experiment was performed at least three times (*n* = 3). The statistical significance of the difference between means (*p* ≤ 0.05 considered significant) was evaluated by one-way analysis of variance (ANOVA) and Tukey tests in XLTAT software version 2018.1.

## Results and discussion

The total protein content of the initial biomass was 0.7 g protein g^−1^ dry weight, a value close to published values for *Spirulina* obtained in several studies (Ahda et al. 2023; Kurpan et al. 2024)*.* The C–PC content was 0.24 g C–PC g^−1^ dry weight, corresponding to 34.2% of total protein content. This value is consistent with the findings of a previous study (Athiyappan et al. 2024) reporting a C–PC content in *Spirulina* of about 25% of total dry biomass*.* Non-pigmented proteins accounted for 65.8% of total protein, which is equivalent to 0.46 g protein g^−1^ dry weight of the *Spirulina sp.* studied.

## Influence of ultrasound parameters on protein recovery

**Fig. 3:**
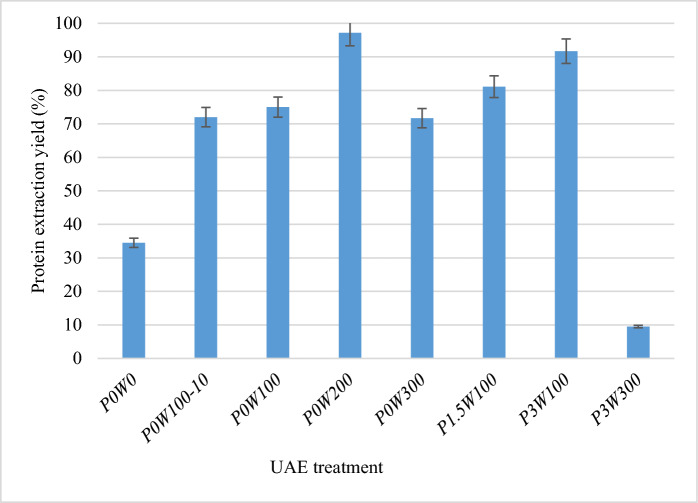
Differences in total protein extraction yield between different UAE conditions. The results shown are the mean values for three experiments ± SD (*n* = 3)

*Spirulina* has a cell wall consisting principally of murein (peptidoglycan), with no cellulose (Machado et al. 2022). A blank reference was established to facilitate evaluation of the true effect of ultrasound. In the baseline experimental conditions used to establish this reference, with a stirring rate of 500 rpm and no cell disruption, the soluble protein extraction yield was approximately 34% of the total protein content after 60 min of treatment (P0W0) (Fig. 3).

**Fig. 4:**
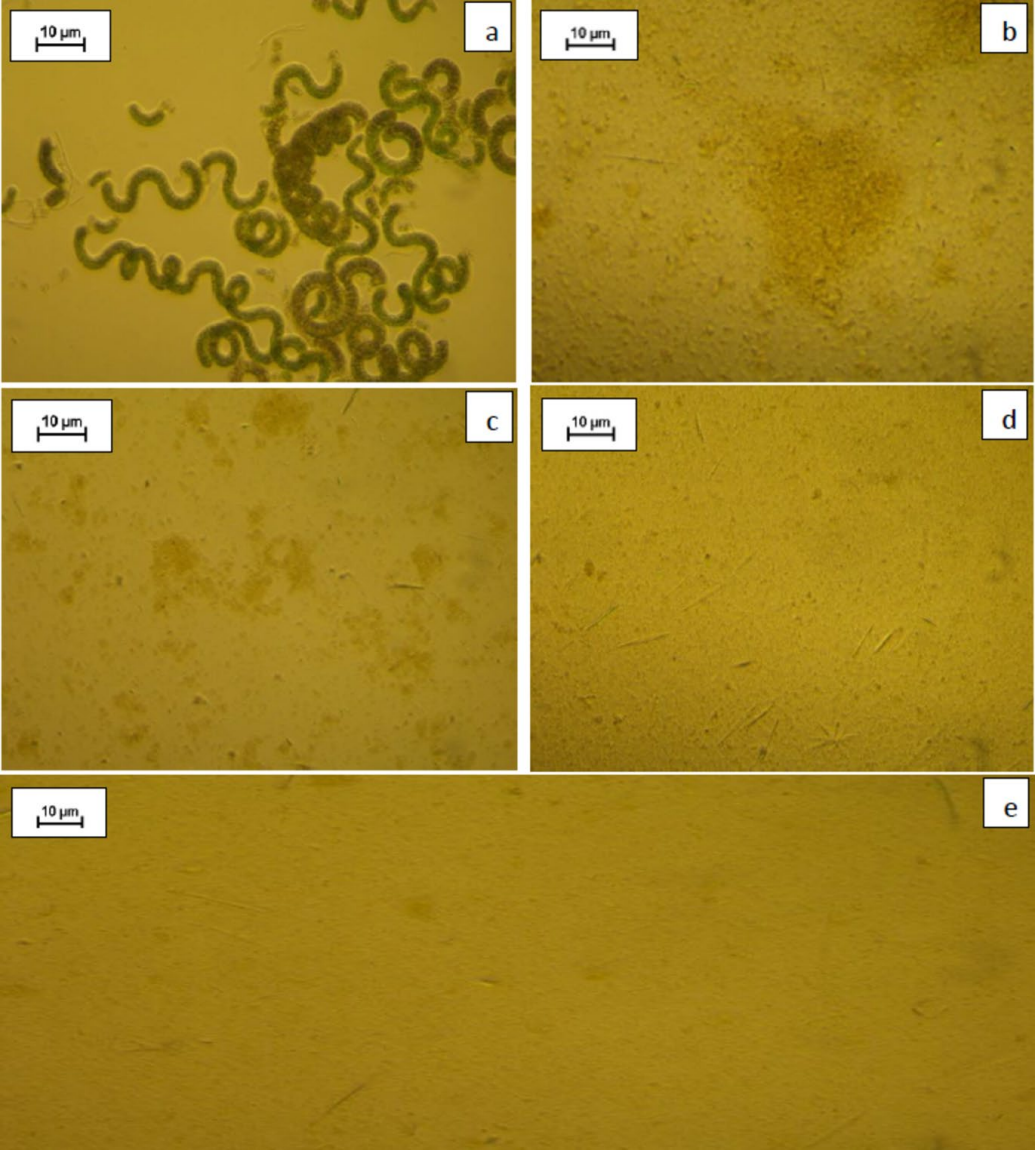
Microscopy observations of fresh *Spirulina sp*. before and after UAE-mediated cell disruption. **a** Before disruption; **b** UAE at 0 bar, 0 W (P0W0); **c** UAE at 0 bar, 100 W (P0W100); **d** UAE at 1.5 bar, 100 W (P1.5W100); **e** UAE at 3 bar, 100 W (P3W100)

Microscopy revealed that stirring damaged the cell wall in this fragile species (Fig. 4** a, b**). Ultrasound parameters had a significant effect on protein extraction yield (*p* < 0.01). The application of a gentle UAE treatment at 100 W for 10 min (experiment P0W100-10) resulted in the release of more than 72% of total protein content into the aqueous medium, whereas only 34.5% (P0W0) of total protein content was released in the absence of UAE treatment (Fig. 3). Microscopy revealed that the cells were completely disrupted and lost their spiral shape during UAE (Fig. 4c). Moreover, the extraction yield at atmospheric pressure increased to 97.2% if the power was increased to 200 W (P0W200), whereas it decreased to 71.7% if the power was increased further to 300 W (P0W300) (Fig. 3). The negative effect of increasing UAE power from 100 to 300 W at a pressure of 3 bar was much more pronounced, with protein yield decreasing sharply, from 75% to 9.5%.

At a power of 100 W, increasing the pressure significantly improved protein extraction yield. Specifically, the yield was 71.5% at atmospheric pressure (P0W100), increased to 81% at 1.5 bar (P1.5W100), and reached 92% at 3 bar (P3W100). Conversely, at a power of 300 W, the yield decreased sharply with increasing pressure, dropping from 72% at atmospheric pressure (P0W300) to only 9.5% at 3 bar (P3W300) (Fig. 3). At atmospheric pressure, the duration of extraction had a minimal effect on protein release. For instance, with extraction times of 10 min (P0W100–10) and 60 min (P0W100), yields of 72% and 75%, respectively were obtained (Fig. 3). In addition, a low-frequency UAE process (P0W200) gave a high protein extraction yield of up to 97% with *Spirulina sp.*. The efficiency of protein release was influenced by the ultrasound parameters used. For the purposes of comparison, simply stirring the cells in the ultrasound apparatus (the blank in this study) resulted in a protein extraction yield of 34%. Protein extraction from cyanobacteria and microalgae remains challenging due to the presence of a cell wall, which acts as a major barrier. Efficient cell disruption methods are required to gain access to intracellular metabolites (Machado et al. 2022). Another challenge in protein extraction from microalgae and cyanobacteria is that the various proteins have diverse intracellular distributions and are found in different cell compartments.

Plasma membrane proteins are generally easy to recover, whereas the extraction of other proteins located at sites deeper within the cells requires more intensive cell disruption (Verdasco-Martín et al. 2019; Giannoglou et al. 2022). The proteins that are not released are assumed to be attached to the thylakoid system and photosynthetic lamellae. A large proportion of the total protein (66%) was not extracted, possibly because not all the cells were lysed, or because the non-released proteins were retained in intracellular structures. High recovery yields (70%), very similar to those obtained after 1 h, were obtained after 10 min of US treatment at a power of 100 W. Ultrasonication can therefore act very rapidly, within 10 min, so it is not necessary to use long extraction times (e.g. 1 h). The efficiency of ultrasound treatment at 100 and 200 W and atmospheric pressure may be attributed to a mechanical effect of US, disrupting the cell walls of *Spirulina* and releasing proteins. The high recovery yields obtained show that UAE can destroy almost all the cellular structures, including the thylakoids, thereby releasing C–PC. Under these conditions, the extraction yield was greater, but the solution obtained was also more complex and darker in color.

Increasing the power to 300 W decreased protein extraction yield, possibly due to the degradation of proteins through physical and mechanical effects. With low-frequency ultrasound, the mechanical effects of the pressure and power of UAE may be the principal drivers of the degradation of large molecules, such as proteins (Liu et al. 2018). The rupture of *Spirulina* cells by UAE involves two mechanisms that alter cell structure: cavitation and acoustic streaming. Cavitation is the production of microbubbles, which implode violently, sending out shock waves that disrupt the surrounding material (Chit et al. 2023).

The protein analysis was performed on soluble proteins, so aggregates were not characterized. Similar results were obtained in a previous study (Zhou et al. 2023) in which a high ultrasonic intensity was found to be detrimental to the pigments and proteins. Alternatively, the acoustic power may have been too high, and the creation of a bubble cloud at the sonoprobe surface may have led to acoustic shielding, preventing correct transmission of the ultrasound waves in the tank (Grosjean et al. 2019). Alternatively, the kinetics of protein release may be faster at higher pressures, leading to a denaturation of the proteins, which remain in the liquid phase for longer. This denaturation may be attributed to the mechanical effects of ultrasound or the appearance of free radicals.

At low power (100 W), increasing the pressure from 0 to 3 bar led to an increase in protein extraction yield. Microscopy revealed a strong correlation between pressure and cell disruption (Fig. 4c–e). This result may reflect an increase in the collapse pressure of the bubbles formed during acoustic cavitation, as reported in a previous study (Liu et al. 2022). Increasing the pressure also enhances the diffusion of molecules out of cell residues (Vernès et al. 2019). At high power (300 W), an increase in pressure from 0 to 3 bar resulted in a significant decrease in protein extraction yield, from 71.7 to 9.5%, probably due to the combined effects of high pressure and high power, as discussed above.

## Ultrafiltration

**Fig. 5:**
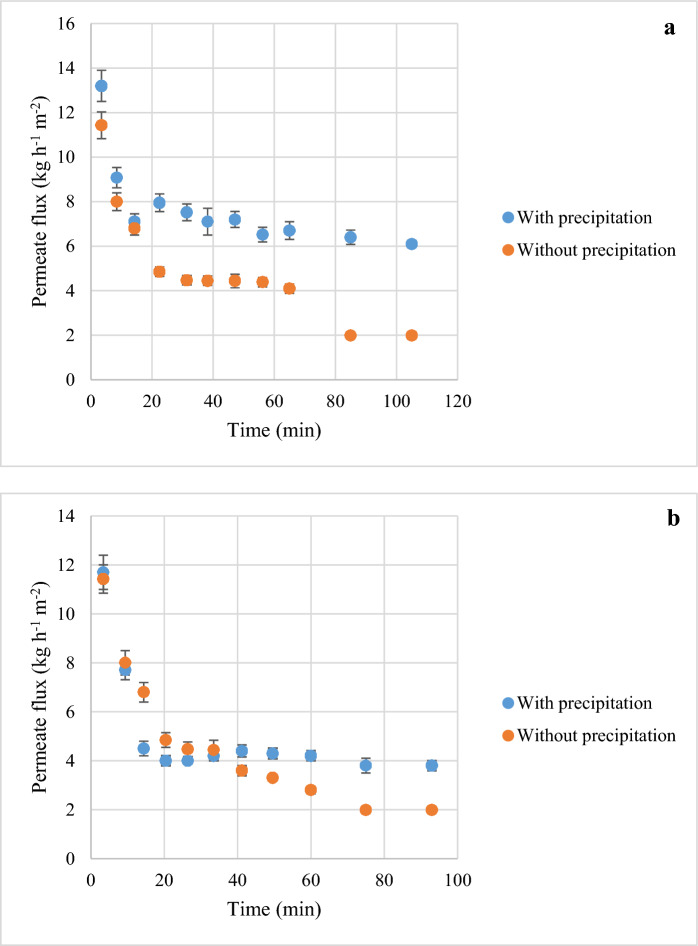
Permeate flux for the crude protein extract subjected to ultrafiltration, at different time points, with and without ammonium sulfate precipitation **a** 100 kDa **b** 10 kDa. Results are expressed as the mean value for the three replicates of each experiment (*n* = 3) ± SD. Blue line: with precipitation; orange line: without precipitation

The supernatant obtained after UAE and centrifugation contained many impurities in addition to proteins: cell debris, lipid residues, and several pigments composed of C–PC, chlorophylls, and carotenoids. Ultrafiltration experiments were performed directly on 100 ml of extract introduced into an Amicon cell, to which a static pressure of 2 bar was applied. The permeate was continuously recovered during filtration, but the volume concentration ratio remained below 1.5 in all cases. The permeate flux decreased sharply from 12 kg h^−1^ m^−2^ to a limiting value of 2 kg h^−1^ m^−2^ after a few min for both the 10 kDa and 100 kDa membranes (Fig. 5).

The limiting flux was similar for both membranes, but the initial flux differed slightly, with a variance of 0.5 (kg h⁻^1^ m⁻^2^) ^2^. The observed decrease in membrane ultrafiltration flux over time was linked to the development of membrane fouling (Xu et al. 2022). The fouling of ultrafiltration membranes is typically primarily influenced by the presence of a cake layer or aggregates (Tanudjaja et al. 2022). Other factors such as the membrane material and operational variables also play a significant role in membrane fouling, as highlighted in a previous study (Marson et al. 2021). This behavior can be explained by the formation of a polarization layer at the start of filtration due to the accumulation of biomolecules on the membrane surface, increasing solute concentration in the medium (Fernández and Riera 2012). This accumulation reduces membrane efficiency, such that permeate flux is no longer controlled by the porosity of the membrane but by this layer (Marshall et al. 1993). According to Darcy’s law, which explains the change in membrane performance over time, the decrease in permeate flux is due to an increase in medium viscosity, provided that the transmembrane pressure applied and membrane resistance are constant (Rida et al. 2024).

**Fig. 6:**
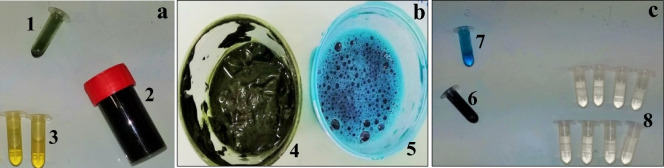
**a** Color of the various compartments after direct UF. (1) UAE crude extract, (2) retentate, (3) permeate. **b** Color of the various compartments after precipitation with ammonium sulfate (25% w/v): (4) pellet, (5) supernatant. **c** Color of the various compartments after ammonium sulfate precipitation followed by UF: (6) Crude extract before precipitation (7) retentate, (8) permeate

The permeate obtained was yellow, indicating the presence of certain pigments, whereas the retentate was very dark (Fig. 6a). The total protein retention rate was 20 ± 1.2% for the 10 kDa membrane and 12 ± 0.8% for the 100 kDa membrane (Table 2). This result confirmed that the rate of protein passage through the membrane increased with increasing membrane MWCO. However, the C–PC retention rate was 92 ± 0.2% for the 100 kDa-cutoff membrane and 94 ± 0.5% for the 10 kDa-cutoff membrane. These results are consistent with those of a previous study (Nisticò et al. 2022) reporting a C–PC retention rate of 96% with a 20 kDa-cutoff PES membrane. The high rate of C–PC retention can be explained by the C–PC in *Spirulina sp*. being present predominantly in the form of trimers (α_3_β_3_) with a mean molecular weight of about 91 kDa, ranging from 81 to 161 kDa depending on the pH. The part lost in the permeate probably corresponds to the α and β subunits, which have molecular weights of 16 and 21 kDa, respectively (Zheng et al. 2019; Amarante et al. 2020). However, this high rate of rejection with the 100 kDa membrane confirmed that the layer created during filtration reduced the permeability of the membrane and altered its selectivity.

**Table 2:** Influence of the extraction treatment on the filtration selectivity of the 10 kDa and 100 kDa MWCO membranes. Initial volume for UF: 100 ml; precipitation performed with 25% ammonium sulfate

	UF without precipitation		UF with precipitation	
	10 kDa	100 kDa	10 kDa	100 kDa
[TP]_i_ (mg protein ml^−1^)	6.5	6.5	6.1	6.1
[TP]_R_ (mg protein ml^−1^)	6.3	6.4	13.7	13.6
[TP]_P_ (mg protein ml^−1^)	5.2	5.7	3.1	3.1
Protein retention rate (%)	20	12	0	0
Mass balance deviation (%)	18	11	3	3
Retentate C–PC recovery (%)	94	92	95	91
Permeate protein recovery (%)	42	48	60	67
Permeate protein content (%)	19	22	28	31

[TP] refers to the total protein concentration of the sample. The results shown are the mean values for three experiments

An analysis of protein mass balance revealed that, in both cases, a significant proportion of the total protein was not recovered in the permeate or the retentate: 18 and 11% of total protein remained on the surface of the 10 kDa and 100 kDa cutoff membranes, respectively (Table 2).

An ammonium sulfate (25% w/v) precipitation step was incorporated into the procedure to improve ultrafiltration efficiency by clarifying the extract before ultrafiltration. This treatment probably removed cell debris, lipid residues, and pigments, as the precipitate was green, whereas the supernatant was blue (Fig. 6b).

**Fig. 7:**
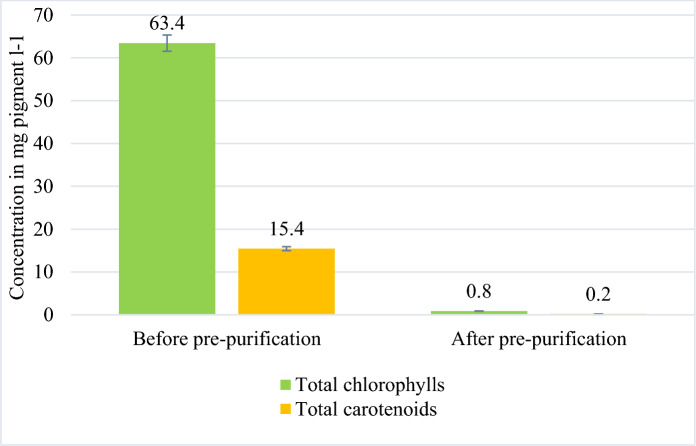
Total chlorophyll and total carotenoid concentrations in the crude protein extract (obtained in experiment P0W200) before and after ammonium sulfate (25% w/v) precipitation. The results shown are the mean values for three experiments ± SD (*n* = 3). Green: Total chlorophylls, Yellow: Total carotenoids

The precipitation step eliminated up to 98 ± 0.1% of carotenoids and chlorophyll from the UAE extract (Fig. 7). The supernatant obtained following the ammonium sulfate precipitation step contained up to 85% of the initially extracted proteins (Table 2), mostly the colorless proteins. This precipitation step increased C–PC content slightly from 40 ± 0.3% to 42 ± 0.5%.

Ammonium sulfate (25% w/v) precipitation of the supernatant increased permeate flux at the end of the ultrafiltration process. The flux stabilized at 6 kg h^−1^ m^−2^ and 4 kg h^−1^ m^−2^ for the 100 kDa and 10 kDa MWCO membranes, respectively; there was therefore a variance of 2 (kg h⁻^1^ m⁻^2^) ^2^ between the two membranes. However, a more significant difference — 24.5 (kg h⁻^1^ m⁻^2^) ^2^ was observed between the two membranes for initial permeate flux. Membrane pore size therefore had a stronger effect on initial permeate flux than on limiting flux. Particle removal decreased membrane fouling, resulting in a greater difference between the two membranes. The 100 and 10 kDa MWCO membranes retained 91 ± 0.5% and 95 ± 1% of C–PC, respectively. It was, therefore, possible to recover a non-pigmented fraction of proteins in a colorless permeate without impurities, pigments, or C–PC (Fig. 6** c**). The rate of C–PC retention after ammonium sulfate precipitation was similar to that before precipitation. In the retentate, total protein concentration increased to 13.7 g protein l^−1^ and C–PC concentration increased to 9 g C–PC l^−1^, corresponding to a content of 65% for a final volume/concentration ratio of 4.

The use of the clarified supernatant decreased the protein retention rate on the two membranes tested, with a 3% fall in mass balance. These results suggest that protein retention in the absence of clarification was linked to the creation of a cake-like structure by impurities on the membrane surface. The removal of these impurities decreased the formation of this cake, leading to a lower level of protein retention, and lower losses at the membrane surface. After precipitation, the retentate was dark blue (Fig. 6), with a high C–PC recovery yield (95%), whereas the permeate was transparent and colorless, with a high protein content, corresponding to the recovery of a large proportion of the non-C–PC proteins.

## Conclusion

This article describes a novel process for valorizing two different protein fractions of *Spirulina platensis*. A total protein recovery yield of up to 97% can be obtained with an ultrasound system, with a frequency of 12 kHz, an ultrasonic power of 200 W, and an aqueous medium, at atmospheric pressure. Increasing the pressure increases recovery yield, whereas increasing power may lead to partial degradation of the protein and, thus, its entrapment in the solid residue. Despite the promising yield, the extracts obtained contained many impurities, including cell fragments and pigment complexes, which significantly limited filtration efficiency. This issue was addressed by introducing an ammonium sulfate (25% w/v) precipitation step, which effectively removed pigments (up to 98% of chlorophylls and carotenoids were removed by this approach) and impurities from the crude protein extracts.

Following this pretreatment, semi-frontal ultrafiltration was conducted, resulting in the recovery of 95% and 91% of the C-phycocyanin in the retentate with 10 and 100 kDa MWCO membranes, respectively, whereas up to 67% of total non-pigmented proteins were found in the permeate with the 100 kDa MWCO membrane. These findings highlight the potential of combining ultrasound and ultrafiltration for microalgae biorefinery applications.

Nevertheless, it is imperative to check the selectivity of the membrane under tangential flow conditions at a larger scale, with the aim of achieving higher permeate fluxes suitable for industrial applications.

## Data Availability

The data supporting the findings of this study are available within the paper.
